# Oxidative Stress and Non-Alcoholic Fatty Liver Disease: Effects of Omega-3 Fatty Acid Supplementation

**DOI:** 10.3390/nu11040872

**Published:** 2019-04-18

**Authors:** Jinchunzi Yang, Marta Fernández-Galilea, Leyre Martínez-Fernández, Pedro González-Muniesa, Adriana Pérez-Chávez, J. Alfredo Martínez, Maria J. Moreno-Aliaga

**Affiliations:** 1 Centre for Nutrition Research, School of Pharmacy and Nutrition, University of Navarra, 31008 Pamplona, Spain; jyang@alumni.unav.es (J.Y.); mfgalilea@unav.es (M.F.-G.); lmartinez.7@alumni.unav.es (L.M.-F.); pgonmun@unav.es (P.G.-M.); aperez.39@alumni.unav.es (A.P.-C.); jalfmtz@unav.es (J.A.M.); 2 Department of Nutrition, Food Science and Physiology, School of Pharmacy and Nutrition, University of Navarra, 31008 Pamplona, Spain; 3 IDISNA, Navarra’s Health Research Institute, 31008 Pamplona, Spain; 4 CIBERobn Physiopathology of Obesity and Nutrition, Centre of Biomedical Research Network, ISCIII, 28029 Madrid, Spain

**Keywords:** oxidative stress, non-alcoholic fatty liver disease, aging, omega-3 fatty acids

## Abstract

Aging is a complex phenomenon characterized by the progressive loss of tissue and organ function. The oxidative-stress theory of aging postulates that age-associated functional losses are due to the accumulation of ROS-induced damage. Liver function impairment and non-alcoholic fatty liver disease (NAFLD) are common among the elderly. NAFLD can progress to non-alcoholic steatohepatitis (NASH) and evolve to hepatic cirrhosis or hepatic carcinoma. Oxidative stress, lipotoxicity, and inflammation play a key role in the progression of NAFLD. A growing body of evidence supports the therapeutic potential of omega-3 polyunsaturated fatty acids (n-3 PUFA), mainly docosahaexenoic (DHA) and eicosapentaenoic acid (EPA), on metabolic diseases based on their antioxidant and anti-inflammatory properties. Here, we performed a systematic review of clinical trials analyzing the efficacy of n-3 PUFA on both systemic oxidative stress and on NAFLD/NASH features in adults. As a matter of fact, it remains controversial whether n-3 PUFA are effective to counteract oxidative stress. On the other hand, data suggest that n-3 PUFA supplementation may be effective in the early stages of NAFLD, but not in patients with more severe NAFLD or NASH. Future perspectives and relevant aspects that should be considered when planning new randomized controlled trials are also discussed.

## 1. Introduction

### 1.1. Aging and Oxidative Stress

Aging is a gradual decline of physiological function with age [1] accompanying the intrinsic, inevitable and irreversible age-related process of loss of viability and increase in vulnerability in human beings [2]. Aging is a ubiquitous complex phenomenon as a consequence of the interaction of genetic, epigenetic, environmental, and stochastic factors throughout life. During this multifactorial process, the damages occurring in molecules, cells, and tissues gradually accumulate in a timely manner. The genetic predisposition and epigenetic modifications afterward have mostly determined the acceleration or the prevention of aging and its related pathophysiological functions at a whole organism level. General statistics have shown that as each individual reaches the mid-age of their biological lifespan, numerous subsequent malign, pathological, and deteriorative consequences start to interfere with the cardiovascular system, metabolic processes, neurodegenerative disorders, muscle and vision functions, among others [3,4,5,6].

Reactive oxygen species (ROS) are generated within the cells by cellular metabolic activities such as cell survival, stressor responses, inflammation [7,8], and environmental factors, such as air pollutants or cigarette smoke [9]. Increased ROS production has been recognized as a critical contributor to aging since the middle 50′s when D. Harman hypothesized “*The free Radical Theory of Aging*” [10] This theory proposed that highly unstable and reactive molecules generated by cellular metabolism or environmental factors cannot be removed or neutralized, which produce proteins, lipids, and DNA damage, eventually leading to a defective function of the organelles in aged subjects. Presently it is known that during the final third of the lifespan, aged animals and humans have lower adaptive homeostatic capabilities [11,12] mainly caused by an impaired antioxidant defense system, which in turn promotes the accumulation of oxidative stress-induced molecular damage and triggers cellular senescence [11]. Oxidative stress can be evaluated by several biomarkers. Hydrogen peroxide, superoxide radical, oxidized glutathione (GSSG), carbonyls, and nitrotyrosine can be easily measured from plasma as biomarkers of oxidation [9]. Lipid peroxidation refers to the oxidative degradation of lipids generally located in biological membranes including phospholipids and cholesterol which propagates free radicals [13]. Quantifying the secondary breakdown products can indicate in vivo lipid peroxidation. Several biomarkers are identified for this: 4-hydroxynonenal (4-HNE), 4-hydroxyhexenal (4-HHE), malondialdehyde (MDA), F2-isoprostanes, α-tocopherol concentration, plasma thiobarbituric acid-reactive substances (TBARS) level, total reactive antioxidant potential (TRAP), superoxide dismutase (SOD) activity and low density lipoprotein (LDL) oxidative susceptibility [13]. Plasma and urine F2-isoprostanes have been identified as excellent and sensitive biomarkers of in vivo lipid peroxidative damage [13,14]. These compounds are formed in a free radical-dependent manner in cell membranes at the site of free radical attack from arachidonic acid [14]. Their production has been reported to be altered in many syndromes putatively associated with oxidative stress [15]. Moreover, human aging has been characterized as a chronic, low-grade inflammation state, widely named as “inflammaging” [16]. Thus, chronic cell oxidative stress activates a pro-inflammatory program leading to acquisition of the senescence-associated secretory phenotype (SASP) characterized by the increased secretion of pro-inflammatory factors involving the secretion of soluble factors (interleukins, chemokines and growth factors), degradative enzymes such as matrix metalloproteases (MMPs) and insoluble proteins/extracellular matrix (ECM) components. Inflammaging, caused by an accumulation of senescent cells exhibiting SASP in tissues, is considered a risk factor for the development of most age-related diseases and therefore, for morbidity and mortality in the elderly [17].

### 1.2. Aging and the Pathophysiology of Non-Alcoholic Fatty Liver Disease (NAFLD)

#### 1.2.1. Concept and Pathogenesis of NAFLD

Liver function and structure impairment is one of the predominant hallmarks of aging [18]. NAFLD is considered to be the consequence of excessive accumulation of triglycerides (TG) in the cytoplasm of hepatocytes (> 0.5%) without the over-consumption of alcohol in order to compensate for the increased cellular content of non-esterified fatty acids (free fatty acids; FFAs) [19]. NAFLD is highly prevalent worldwide [20] and estimated to be globally around 24% according to data published in 2016 [20]. NAFLD is the most frequent cause of chronic liver disease in western countries [16,21,22], encompassing a broad spectrum of physio-pathological conditions from simple steatosis to non-alcoholic steatohepatitis (NASH). According to accepted clinical criteria [23], NASH should only be established when hepatocyte fat storage is accompanied with lobular inflammation and ballooned hepatocytes, where the grade of fibrosis sharpens progressively by stage. If no proper therapeutic approach is implemented, NASH can ultimately progress to hepatic cirrhosis or hepatic carcinoma where the scar tissue becomes irreversible, the liver losses its functions partly or completely, or even develop to hepatocellular carcinoma [24]. NAFLD can trigger or manifest other cardiometabolic disorders including obesity, disrupted glucose and lipid metabolism, insulin resistance (IR) and type 2 Diabetes Mellitus [25,26,27,28]. Aging is also the most common cause for the progression of NAFLD. Indeed NAFLD is commonly found in the elderly, but the results from the Rotterdam study suggested that the prevalence of NAFLD decreased with advancing age, suggesting a positive selection of elderly without NAFLD [29]. Finally, regarding aging and NAFLD, it is important to note that the effect of aging is different between men and women because women experience many physical changes associated with menopause during aging. Although controversial results have been found on the direct cause or on the mechanisms involved, there is increasing evidence indicating that post-menopausal women are more susceptible than men to develop NAFLD [30]. The study of Klair et al. [31] showed that time from menopause is directly associated with an increased likelihood of having more severe NAFLD [31]. In support of this hypothesis, studies in rodents suggested that these observed deleterious aging-related effects on females’ livers might be attributed to impaired lipid metabolism. In fact, in these models, estrogens increased fatty acid β-oxidation by AMPK activation [32,33]. Taking together these studies, it could be speculated that in menopausal women, lower serum estrogen levels might constitute a major cause for impaired lipid accumulation/break down balance and therefore for the promotion of NAFLD development. However, contrarily, the study of Veronese et al. [34] described that the years from menopause are not associated with the severity of NAFLD. Moreover, the study found that adiposity and metabolic syndrome features were associated with higher liver steatosis levels, concluding that not menopause itself but the menopause-associated increased adiposity (particularly the excessive accumulation of abdominal adiposity) is the major cause for NAFLD development in post-menopausal women [34].

However, the pathogenesis of liver disease during aging remains inaccurately defined. Nonetheless, aging is accompanied by a gradual decrease of hepatic blood flow and liver volume ranging from 20% to 40% [18,35,36]. In addition to understanding the nature of these changes, few studies conducted in humans have investigated cholesterol levels. Studies in humans have shown a decrease in low-density lipoprotein by 35% [37,38] and an increase in biliary cholesterol output, possibly related to a decrease in cholesterol degradation to bile acid [39].

NAFLD has a complex pathophysiology with multiple manifestations/complications. Liver steatosis arises as a consequence of an imbalance between hepatic lipid accumulation (from accelerated FFA influx and de novo lipid synthesis) and hepatic lipid clearance (free fatty acid oxidation (FAO) and very low-density lipoprotein (VLDL) excretion) [40]. In terms of pathogenesis of NAFLD, a “two hits hypothesis” was proposed over two decades ago [41] to make an approach to unravel the progression from NAFLD to NASH. The “first hit” core is peripheral IR as a leading cause accompanying obesity and metabolic syndrome. In adipose tissue (mainly visceral depot), IR causes an increase in lipolysis, and consequently the delivery of FFA into the liver increases and TG accumulation occurs. Moreover, de novo lipogenesis (DNL) plays a pivotal role in FFA synthesis, and the excess of carbohydrates is converted into fatty acids and esterified into TG [42]. In contrast, the export of FFA and TG is decreased, while the beta-oxidation of mitochondrial long-chain fatty acids is increased. Indeed, it has been proposed that hepatic TG accumulation is also probably a consequence of saturation of FFA oxidation and VLDL) secretion, since both of these pathways are up-regulated rather than decreased in patients with NAFLD [43]. However, several studies have suggested a protective effect of TG [44,45] and that an increased level of intrahepatic TG may be a biomarker instead of a cause factor of IR [46].

A “second hit” seems to be needed to develop NASH from NAFLD, such as oxidative stress, which in terms may explain the progression to liver fibrosis. An imbalanced production of reactive nitrogen species (RNS) or ROS and the antioxidant molecules of the organism produces oxidative stress, which can induce hepatocellular injury by the inhibition of the mitochondrial respiratory chain enzymes and the inactivation of both glyceraldehyde-3-phosphate dehydrogenase and membrane sodium channels. Furthermore, ROS cause lipid peroxidation and cytokine production, contributing to hepatocellular injury and fibrosis [47], and promote the progression from simple steatosis to NASH [48]. Furthermore, ROS induce the directional migration of resident hepatic pro-fibrogenic cells, resulting in liver fibrosis [49].

**Figure 1:**
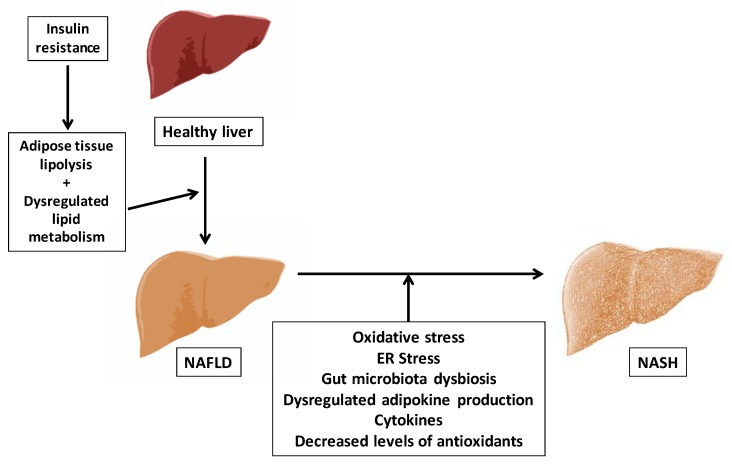
Multiple parallel hits NAFLD pathogenesis model. Non-alcoholic fatty liver disease (NAFLD). Non-alcoholic steatohepatitis (NASH). Endoplasmic reticulum (ER).

In the year 2010, Tilg and Moschen [50] proposed a multiple parallel hits model, suggesting that many hits may act in parallel favoring the progression from NAFLD to NASH, finally resulting in liver inflammation and endoplasmic reticulum (ER) stress, and that gut and adipose tissue derived factors may play a central role (Figure 1).

Lipotoxicity seems to play a key role in the pathophysiology of NASH. Lipotoxic injury is also triggered by an excessive FFA flux, especially of saturated fatty acids (SFAs), rather than due to simple TG accumulation. The excess of FFA facilitates the generation of lipotoxic metabolites (such as ceramides, diacylglycerol, lysophosphatidyl choline and ROS) contributing also to the development of liver oxidative stress [43]. Moreover, in NAFLD and aging, increased visceral adipose tissue is associated with increased infiltration of M1 macrophages, which triggers adipose tissue IR and inflammation and leads to a disturbed adipokine profile (low adiponectin, an anti-inflammatory adipokine with beneficial effects in NAFLD; and high levels of pro-inflammatory adipokines such as leptin, IL-1β, IL-6 and tumor necrosis factor-α; TNF-α), which finally induces liver inflammation and hepatic IR, as previously stated [51]. Therefore, it is clear that signals and hormones derived from the adipose tissue beyond toxic lipids might play a central role in NAFLD/NASH.

In the crosstalk between the gut and the liver, the modifications in the microbiota are considered to have major influence in NASH progression [50]. The human gut is colonized by at least 100 trillion of microorganisms (gut microbiota) that maintain symbiotic relationships with the host. Several lines of evidence have demonstrated strong relationships between changes in gut microbiota composition and the etiology of obesity, inflammation, type 2 diabetes and NAFLD [52]. A major mechanism linking gut dysbiosis with the progression of chronic liver diseases is the translocation of bacteria or bacterial products into the portal circulation [53]. The gut bacteria may contribute to NAFLD by producing bacteria-derived endotoxins (lipopolysaccharide, LPS). It has been shown that feeding with a high-fat or a high-carbohydrate diet causes elevated levels of circulating endotoxin [54], which may also affect the accumulation of hepatic fat [50]. The gut microbiota also produces microbial metabolites such as short-chain fatty acids (SCFAs) which play a critical role in regulating host energy harvest [55]. SCFAs have anti-inflammatory properties, can directly act as lipid precursors in the liver, and may act by interacting with the G protein– coupled receptor 43 (Gpr43) [56]. An altered microbiota also inhibits the synthesis of fasting-induced adipocyte factor (FIAF; also known as angiopoietin-related protein 4, ANGPTL4), which has been associated with a higher accumulation of lipids in the liver [53]. Moreover, the endogenous ethanol produced by the intestinal microbiota favors the transport of endotoxins in the gut vessels [57]. It has been observed that patients with NASH have higher abundance of ethanol-producing bacteria in their gut microbiome, suggesting a potential involvement of alcohol-producing gut microbiota in NASH progression [58]. Ethanol and its derived compounds (acetaldehyde and acetate) [59] induce the formation of ROS by hepatic stellate cells and Kupffer cells [60]. Jones and Neish [61] suggested that the interaction between the gut epithelia and some groups of enteric commensal bacteria induces rapid generation of ROS within host cells [61], and together with LPS, ROS promote increased TLR4 gene expression [62]. Recent data have shown that the gut microbiota regulate the metabolism of the major intracellular antioxidant, glutathione (GSH), in the host organism [63]. Thus, lower levels of GSH can contribute to oxidative stress [64,65].

In conclusion, this multiple parallel hits model reflects more deeply the phenomena that contribute to the pathogenesis of NAFLD/NASH than the previous two hits model. Moreover, aging is an important determinant for NAFLD/NASH development. All the previously described multifactorial mechanisms involved in the pathogenesis of NAFLD/NASH (IR, increased visceral adiposity, inflammation, oxidative and ER stress, dysbiosis) are accentuated during aging, which could be underlying the higher prevalence of these pathologies among elderly. Thus, several therapeutic approaches can be merged from targeting these key factors for the promotion of liver health in the context of aging.

#### 1.2.2. Genetic Variances Susceptibility for NASH and Oxidative Stress

NAFLD is also considered as a poligenic disease [66] where different polymorphisms can affect the three main pathways: genes that participate in TG accumulation, in inflammatory processes and that are part of the oxidative-stress pathway. (i) Genes that participate in the accumulation of triglycerides such as: patatin-like phospholipase domain containing protein 3 (PNPLA3) and transmembrane 6 superfamily member 2 (TM6SF2) [67]. PNPLA3 rs738409 polymorphism has been recently associated through a meta-analysis with NAFLD susceptibility and also to other aggressive diseases [68]. In addition, Li et al. [69] have found a significant association of TM6SF2 rs58542926 polymorphism and the risk of NAFLD. (ii) Genes involved in the inflammatory processes: TNF, IL-6 and toll-like receptor 4 (TLR4) [66]. TNF-238 [70,71] and TNF-308 [72] are the main polymorphisms of TNF related with the disease, although their roles are still under consideration. -174 polymorphism in the IL-6 gene promoter region has been shown to be more prevalent in NAFLD patients than in healthy subjects [71,72]. In the case of TLR4, -299 polymorphism seems to be preventive [73], although a polymorphism (-159) in its co-receptor CD14 has been associated with an increased risk of NAFLD [74]. (iii) Genes that are part of the oxidative-stress pathways: SOD2 gene (mitochondrial enzyme manganese-dependent superoxide dismutase (MnSOD) is encoded by this gene), uncoupling protein 3 (UCP3) and glutamate—cystein ligase (GCLC) [66]. C47T polymorphism in the SOD2 gene has been associated with NASH in obese children [75] and with NAFLD fibrosis severity [76]. A UCP3 single nucleotide polymorphism (SNP) has been related with NAFLD in Chinese children [77] and in obese adults [78]. Furthermore, the genetic variant -129C/T in the GCLC gene has been associated with NASH [79,80].

#### 1.2.3. The Diagnosis of NAFLD

The diagnosis of NAFLD is often based on the following criteria: non-alcoholic, detection of steatosis either by imaging or by histology and an appropriate exclusion of other liver diseases [27,81,82,83]. Daily alcohol consumption approximately below 30 g in men and 20 g in women can be included in the target population according to conducted epidemiological studies [23], but no consensus of the exact quantity has been reached. Ultrasonography (US) and magnetic resonance imaging (MRI) are currently the most employed methods to diagnose NAFLD, since they are non-invasive, comparatively low cost, and commonly available, with a sensitivity of 89% and 77%, and a specificity of 93% and 89% in diagnosing liver steatosis and liver steatohepatitis respectively [84,85]. However, the US is subjective and operator-dependent and not that good at assessing liver steatosis, while the MRI is more objective and better at quantifying steatosis [23]. Liver biopsy is considered to be the “gold” standard for identifying steatohepatitis (NASH) by correlating histological features [86]. Although liver biopsy is the definitive tool to diagnose NASH, it lacks availability being an expensive and invasive procedure, and it would not be necessary to apply since the prevalence of liver steatosis is much more frequent than NASH. Recently, several non-invasive, simple diagnostic indices have shown their utility in diagnosing NAFLD and NASH, since they are calculated based on anthropometric data and biochemical analysis (Table 1). These indices include: the Fatty liver index (FLI) [87,88], the hepatic steatosis index (HSI) [89] and the ZJU (Zhejiang University) index [90]. Other non-invasive scores are the NAFLD fibrosis score [91,92,93] the BARD score [91] and the FIB-4 index [94]. More research is needed to identify and confirm truly independent and quantitative markers of steatosis.

## 2. Nutrients, Oxidative Stress and NAFLD/NASH

Considering the role of oxidative stress in the progression of aging and especially in the risk of developing aging-related pathologies, it is important for researchers to look for potential therapeutic targets that might delay the progression of oxidative damage in the early stages of aging. For this reason, increasing attention has been paid to the role that certain dietary nutrients may play in oxidative stress. Oxidative stress has been identified as a key factor associating obesity with related disorders such as cardiovascular diseases or type 2 diabetes mellitus, which can be triggered by a high-level consumption of several macronutrients: glucose, SFAs or omega-6 polyunsaturated fatty acids (n-6 PUFA) inducing inflammation through nuclear factor kappa-light-chain-enhancer of activated B cells (NF-κB) mediated pathways [102]. Contrariwise a large body of research has investigated the potential beneficial effect of dietary antioxidants such as vitamin A, vitamin E, vitamin C, selenium, α-lipoic acid, resveratrol and other polyphenols in the prevention of metabolic and age-related chronic diseases [103,104,105].

According to previous studies, omega-3 polyunsaturated fatty acids (n-3 PUFA) may exert a protective effect on cardiovascular and metabolic diseases, which have been related to its antioxidant and anti-inflammatory properties [106,107,108]. Richard et al. [109] reported that n-3 PUFA may indirectly act as antioxidants by lowering ROS production and superoxide scavenging. Petinelli et al. [110] found that the liver of NAFLD patients exhibits a marked enhancement in n-6 PUFA/n-3 PUFA ratio, which may favor lipid synthesis over oxidation and secretion, leading to steatosis. In this context, n-3 PUFA have been involved in the regulation of the metabolic switch from anabolism (lipogenesis) to catabolism (FAO) by inhibiting Sterol regulatory element-binding protein 1 (SREBP1c) and activating peroxisome proliferator-activated receptor alpha (PPARα), a positive regulator of FAO [110,111,112]. However, it remains the theoretical concern that the many double bonds of eicosapentaenoic acid (EPA) and docosahexaenoic acid (DHA) may lead to an increased unsaturation index once they are incorporated into the membranes and lipoproteins [113]. This situation could induce higher lipid peroxidation based on the premise that fatty acid oxidizability might be directly associated with the number of double bonds in the fatty acid chain [114]. However, some in vitro and in vivo studies have challenged these theoretical considerations. Indeed, n-3 PUFA may also alleviate IR, lipid accumulation, pro-inflammatory actions, and ROS production, and promote the FAO in NAFLD, in part by modulating the production of bioactive adipokines (leptin and adiponectin) that control the crosstalk between adipose tissue and key metabolic organs such as the liver and muscle [115].

On the other hand, understanding that nutrient intake can also promote or reduce the pro-inflammatory response and oxidative stress occurring in NAFLD and NASH is of crucial importance in order to prevent the development and/or severity of these pathologies [116]. Few studies have suggested that over-consumption of carbohydrate-enriched drinks, which contain elevated levels of fructose increased SREBP1c and downstream fatty acid synthesis genes, resulting in reduced liver insulin signaling [117]. Meanwhile, fructose promotes inflammation by modelling inflammatory genes [118,119,120], and down-regulates the hepatic mitochondria beta-oxidation [121]. Overloaded iron levels and decreased copper level have been suggested to play a role in NASH development, inducing inflammation and oxidative stress through several cellular mechanisms [122,123,124]. Increased dietary intake of SFAs, cholesterol and trans-fat induces DNL leading to ER stress and apoptosis [125]. These nutrients are mostly in favor of the NASH development since they trigger inflammation and ER stress.

In conclusion, increased fructose, SFA, cholesterol, trans-fat, iron intake and decreased copper intake can induce oxidative stress in NAFLD; on the contrary, nutrient intake such as n-3 PUFA, vitamin A, vitamin E, vitamin C, selenium, α-lipoic acid, resveratrol, and other polyphenols may ameliorate oxidative stress and NAFLD. To our knowledge, there are not systematic reviews analyzing the main outcomes of randomized controlled trials aimed to characterize the actions of n-3 PUFA supplementation on oxidative-stress biomarkers.

On the other hand, although several trials and recent meta-analysis have suggested that n-3 PUFA may have beneficial effects on NAFLD patients, the outcomes on different biomarkers of fatty liver disease (AST, ALT, GGT or liver fat content by US) are sometimes heterogeneous [126,127,128,129,130]. Moreover, recent randomized controlled trials have produced conflicting data in NAFLD/NASH patients [131].

Based on these premises, the aims of the current study are to perform a systematic review of the current clinical trials analyzing the potential beneficial effects of n-3 PUFA supplementation on systemic oxidative stress as well as their efficacy in the treatment of NAFLD and NASH in adult patients.

## 3. *n*-3 PUFA in Oxidative Stress and NAFLD/NASH

### 3.1. n-3 PUFA (Dietary Sources and Metabolism)

α-linolenic acid (ALA) and linoleic acid (LA) are essential PUFA obtained from diet, since they cannot be synthesized in humans [26,27]. LA is the major n-6 PUFA, and ALA is an n-3 PUFA [90]. n-6 PUFA can be obtained mainly from poultry, eggs, nuts, sesame seeds, dairy products, and lower amounts in grains and seeds [26,27]. ALA (18:3 n-3) can be found in flaxseed, canola and soybean oils, green leafy vegetables and walnuts [132]. In the body, LA is metabolized to arachidonic acid (AA; 20:4 n-6), and ALA is metabolized to EPA (20:5 n-3) and DHA (22:6 n-3) [102]. The conversion of ALA to EPA is limited; however, some studies show that ALA can be converted to EPA when adequate intake of ALA is ensured and low intake of n-6 PUFA happens. Moreover, in vivo the conversion of ALA to DHA has been reported to be even more limited [133]. Regular intake of EPA and DHA from our diet is needed to guarantee an optimal supply of n−3 PUFA. EPA and DHA are acquired mainly from marine products: fatty fishes such as salmon, tuna, and sardines, which store fatty acids throughout their body, and lean fishes such as cod and hake that store fatty acids in the liver [134]. The common minimal recommended daily intake of n-3 PUFA ranges approximately from 0.35 to 0.40 g per day (0.5% of total fat) [135]. The American Heart Association guidelines suggests a two-portion of fatty fish intake per week to prevent hypertriglyceridemia and cardiovascular disease [136]. Yet, no consensus of the precise amount has been reached. When humans consume EPA and DHA, they partially replace the n-6 PUFA (particularly AA) in cell membranes, especially those of platelets, erythrocytes, neutrophils, monocytes, and liver cells [137]. As a result, ingestion of EPA and DHA from fish or fish oil ameliorates the pro-inflammatory, atherogenic pro-aggregatory and pro-thrombotic effects caused by n-6 PUFA, which leads to down regulation of pro-inflammatory metabolites such as prostaglandin E2, thromboxane A2 and leukotriene B4 [100,138,139].

Western diets usually contain higher amounts of n-6 PUFA and lower amounts of n-3 PUFA (n-6/n-3 PUFA ratio around 15:1). Current evidence suggests that it is imperative to maintain an optimal n-6/n-3 PUFA ratio [140,141,142], since this not only plays a role in the pathogenesis of CVD, but also has an impact on cancer, inflammatory and autoimmune diseases [140]. A suggested ratio of n-6/n-3 PUFA is 3-4:1 to maintain a pro-inflammatory/anti-inflammatory equilibrium in the organism [143,144]. The increase in the n-6/n-3 PUFA ratio can also exacerbate the risk of obesity and NAFLD [145,146,147].

EPA and DHA have been demonstrated to serve as substrates for the formation of a novel series of specialized pro-resolving lipid mediators (SPMs). These SPMs include: EPA derived E-series Resolvins (RvE-1), DHA derived D-series Resolvins (RvD1-6), protectins (NPD1, PDX) and Maresin (MaR1-2) [148]. Studies have demonstrated their potent anti-inflammatory and pro-resolutive properties acting at doses much lower than their n-3 PUFA precursors [108]. Liver inflammation can be resolved by a shift to M2 macrophages, and these SPMs can act as ‘stop signals’ of the inflammatory response and promote liver regeneration [149]. In this way, several studies in obese mice with IR and liver steatosis have found that treatment with some of these SPMs including RvE1, RvD1, 17-HDHA and Maresin 1 decrease adipose tissue and liver inflammation and significantly improve IR and reduce liver steatosis [150,151,152,153,154]. Moreover, n-3 PUFA supplementation from marine sources to obese mice promotes the synthesis of n-3 PUFA-derived SPMs in liver and adipose tissue, ameliorating tissue inflammation and peripheral inflammation and IR [155,156,157]. Interestingly, a clinical trial conducted in humans has shown that short-term n-3 PUFA supplementation for 5 days results in concentrations of SPMs that are biologically active in healthy humans [153]. Other trials in severely obese subjects have demonstrated that the production of anti-inflammatory-pro-resolving lipid mediators in adipose tissue is enhanced after n-3 PUFA treatment [158]. All these findings suggest that the beneficial metabolic effects attributed to n-3 PUFA can be partly mediated by the production of these SPMs in key metabolic tissue such as liver and adipose tissue.

### 3.2. n-3 PUFA and Oxidative Stress

**Figure 2:**
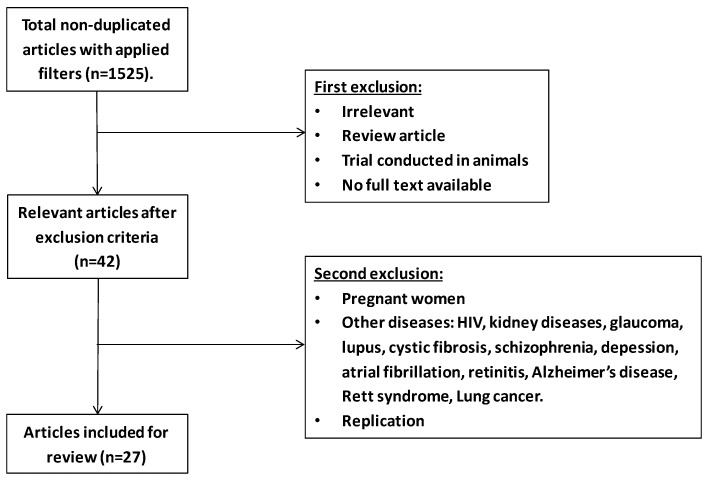
Flowchart of selection process based on n-3 PUFA and oxidative stress.

To perform a review about the effects of n-3 PUFA supplementation on oxidative-stress biomarkers in adults, potentially relevant studies were retrieved by a systematic search in the PubMed database. The search was performed using the terms “n-3 PUFA and oxidative stress”. Around 1525 non-duplicated entries were found. Titles and abstracts were reviewed for the first selection, and full texts were checked only when abstracts itself were not able to reveal the nature of the studies. The filters applied were clinical trial, free full text, human, adults with cardiometabolic disorders, chronic inflammatory diseases, or healthy adults. Twenty-seven studies were included after two selections (Figure 2).

Table 2 summarizes the characteristics of the trials (population, design, intervention) and the main outcomes observed in biomarkers of oxidative stress after n-3 PUFA supplementation in adults.

The studies described in Table 2 include different populations as healthy and overweight/obese adults, patients of both genders with cardiometabolic disorders, metabolic syndrome, T2DM, dyslipidemia, hypertriglyceridemia, with a wide age range. Moreover, doses and supplementation periods as well as the oxidative-stress biomarkers assessed (including urine F2-isoprostanes, 4-HHE and 4-HNE, oxidized LDL, plasma α-tocopherol, and enzymatic activity of Glutathione reductase (GR), Glutathione peroxidase (GPx), and Catalase (CAT) vary from study to study. Regarding the main outcomes, several studies showed a lowering effect on oxidative stress and lipid peroxidation biomarkers with EPA or DHA supplementation [159,160,161,162,163,164,165,166,167,168,169]. However, other studies showed increased levels of oxidative stress or lipid peroxidation biomarkers [170,171,172,173,174,175] or no changes [176,177,178,179,180,181,182,183]. Other studies have observed divergent effects of n-3 PUFA supplementation on different oxidative-stress biomarkers [184,185].

In addition to quantifying the oxidative stress produced by free radicals, several molecules including lipids, DNA and proteins were considered as the main biomarkers since they could be modified by excessive ROS in the microenvironment [186]. Most of the 27 studies above measured the lipid oxidation and its products since they were considered as the widely used biomarker of oxidative stress.

F2-isoprostane is a chemically stable prostaglandin-like isomer generated by the reaction of polyunsaturated fatty acids in membrane phospholipids and free radicals or ROS [187,188,189,190]. F2-isoprostane has been considered as a gold standard lipid peroxidation marker and was identified as an excellent and sensitive biomarker of in vivo lipid peroxidative damage. Indeed, F2-isoprostanes have been found to be elevated in syndromes putatively associated with oxidative stress and aging such as type 2 diabetes, Alzheimer’s disease and NAFLD [13,14,15]. After n-3 PUFA supplementation, several clinical trials have reported decreased urine and/or plasma F2-isoprostane levels in humans [159,160,162,164,166,168]. However, other trials have found no effects on F2-isoprostanes [179,181,182,183,185]. Most of the trials have been performed using n-3 PUFA preparations that contain both EPA and DHA. A few trials performed comparative studies on the effects of EPA and DHA themselves. In this context, Mori et al. [162] demonstrated that both purified EPA and DHA equally reduced urine F2-isoprostanes, suggesting that at least in the short term, the inclusion of regular fish meals providing n-3 PUFA or the supplementation with purified EPA and DHA, can reduce in vivo oxidative stress in humans. Similar outcomes were found in a trial with overweight subjects, mildly hyperlipidemic men, treated with purified EPA or DHA for 6 weeks [164].

Oxidized LDL (oxLDL) levels have been also used as biomarkers of oxidative stress since, low-density lipoproteins can undergo oxidative modification. Regarding the effects of n-3 PUFA supplementation on the oxLDL as an oxidative-stress biomarker, some of them found no significant changes [176,177,178,180], while others revealed that oxLDL susceptibility increased [171,173]. It should be mentioned that the use of oxidized LDL as a biomarker of oxidative stress has been criticized and that the inconsistency of the results obtained in the different trials may be due to the heterogeneity of oxidation products, the low specificity of the antibodies and the different results obtained depending on the assay used [191,192,193].

Malondialdehyde (MDA) is an end-product of lipid peroxidation of polyunsaturated fatty acids including AA. Circulating MDA is one of the most commonly and widely used biomarkers of oxidative stress [194]. As reported for other oxidative-stress biomarkers, the role of n-3 PUFA supplementation on MDA plasma levels remains unclear. Thus, some trials observed that after n-3 PUFA supplementation, MDA plasma levels decreased [160,168]. Other studies demonstrated increased levels of MDA in plasma [170,172,173,175,184]. Various analytical methods have been used to measure MDA in biological samples. The most common is the Thiobarbituric acid reactive substances (TBARS) method [195]. In a study conducted by Higdon et al. [160], MDA level decreased while TBARS level increased. In this regard, TBARS lacks specificity, as many chemically reactive carbonyl groups-containing compounds from different classes of substances including oxidized polyunsaturated fatty acids and carbohydrates, from endogenous sources and foods present in body fluids, can react with TBA. Therefore, it should be taken into consideration that results might vary between studies carried out by TBARS or other more specific techniques for the measurement of MDA [195].

At the clinical level, other biomarkers of oxidative stress analyzed were those related to antioxidant defenses such as CAT, GR, GPx and Heme oxygenase 2 (HMOX2) enzymes [123], plasma GSH and α-tocopherol levels, and total antioxidant capacity [167,179,182,183]. A few studies evaluated the effects of n-3 PUFA on these biomarkers and although some of these showed improved antioxidant defenses levels or total antioxidant capacity [165,167,169], others described neutral effects [179,180,182,183].

The mechanism by which oxidative stress could be reduced following n-3 PUFA supplementation is still unresolved, but it has been assumed that these effects may occur through immunomodulation and a decreased leukocyte activation [196]. In this sense, it is known that activated immune cells produce cytokines (i.e., TNF-α or IL-6) that consequently promote ROS generation [174,197,198]. Interestingly, numerous studies have demonstrated the ability of n-3 PUFA to diminish pro-inflammatory cytokine production [108]. Moreover, it has been proposed that EPA and DHA are more effective acting as antioxidants and as superoxide scavengers in an unsaturation-dependent manner, given the high unsaturation level of n-3 PUFA [109]. Additionally, EPA and DHA can replace AA in cell membranes decreasing AA concentration [199], which serves as a precursor of F2-isoprostanes.

As a matter of fact, it remains controversial whether n-3 PUFA are effective to counteract oxidative stress in humans. Indeed, the heterogeneity in population, treatment duration, doses, and methods to assess oxidative stress in the trials reviewed prevent any definitive conclusion.

### 3.3. n-3 PUFA Supplementation in NAFLD and NASH Adult Patients

**Figure 3:**
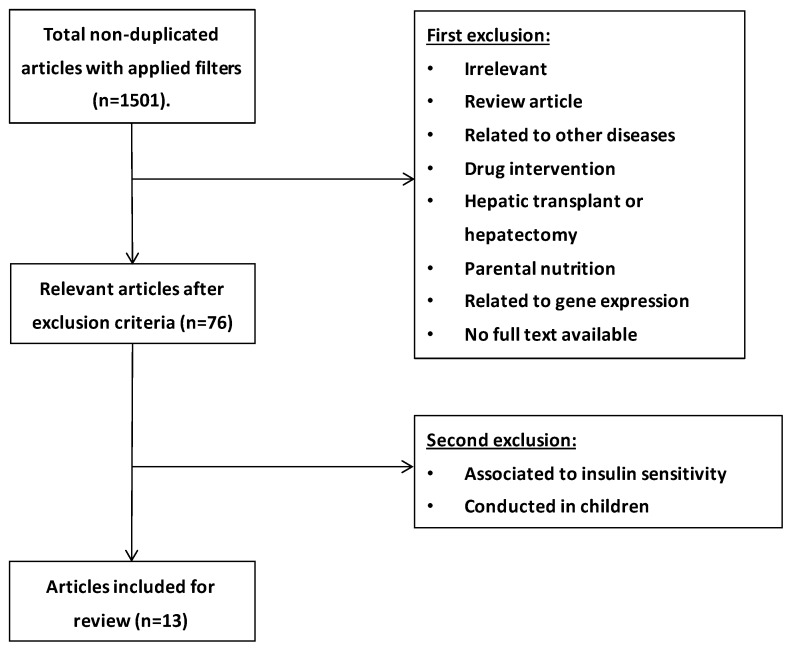
Flowchart of selection process based on n-3 PUFA and NAFLD/NASH.

To evaluate the potential beneficial effect of supplementing n-3 PUFA (including DHA and EPA) in NAFLD and NASH, another systematic review of clinical trials carried out in adults was performed. Potentially relevant studies were retrieved by a systematic search in the PubMed database. The following search terms were used: “n-3 PUFA and NAFLD”, “DHA and NAFLD”, “EPA and NAFLD”, “n-3 PUFA and NASH”, “DHA and NASH”, “EPA and NASH”. Articles were filtered by clinical trials (article type), full text (availability), 10 years (publication date), and only human trials were included. Studies which recruited obese adults (>18 years old) with pre-diagnosed NAFLD at different stages and with liver or blood profile inflammation and oxidative stress related biomarkers were included, as well as studies in patients with NAFLD or NASH associated with diabetes and hyperlipidemia. Studies including a specific ethnic population, NAFLD or NASH associated with cardiovascular diseases or risk factors, polycystic ovary syndrome, and genotypes among others were excluded. Around 1501 non-duplicated entries, with the applied filters of “clinical trials” and “human” were found in the PubMed database. Titles and abstracts were reviewed for the first selection, and full texts were checked only when abstracts themselves were not able to reveal the nature of the studies. Finally, 13 clinical trials were included (Figure 3).

Table 3 describes the characteristics (population, design, etc.) of the selected trials as well as the main findings concerning the effects of n-3 PUFA supplementation in adult patients with NAFLD and NASH.

In 2006, Capanni et al. [200] carried out the first clinical trial in humans to test the efficacy of long-term supplementation of n-3 PUFA in NAFLD. This study in patients with NAFLD confirmed by US showed that oral intake of n-3 PUFA (EPA and DHA in a 0.9/1.5 ratio) did not change BMI, but significantly decreased serum TG and glucose levels in parallel with a reduction in arachidonate and n-6/n-3 ratio. Concerning liver function, the study also revealed that the n-3 PUFA group exhibited lower levels of circulating AST, ALT, and GGT. Moreover, the US and duplex Doppler assays revealed that n-3 PUFA supplementation significantly improved liver echo-texture with a regression of hepatic brightness and higher Doppler perfusion index (DPI), which indicates an improvement in liver blood flow due to intrahepatic fat reduction [200].

The randomized/open-label trial by Spadaro et al. [111] showed similar patterns to the Capanni et al. study [200]. Patients with proven NAFLD by US followed dietary recommendations in concordance with the American Heart Association (AHA) guidelines, with a caloric restriction of 25-30 kcal/kg per day for 6 months. The group supplemented with n-3 PUFA (1 g twice a day) showed decreased circulating levels of TG and increased HDL levels. Moreover, in the n-3 PUFA group, ALT and GGT serum levels were markedly decreased. A complete steatosis regression was observed in 33.4% of patients, and an overall reduction in 50%, suggesting a beneficial effect of long-term n-3 PUFA supplementation to reduce fatty liver. n-3 PUFA supplementation also reduced the inflammatory marker TNF-α and the IR index assessed by HOMA-IR. A correlation between both factors was observed [212]. Thus, TNF-α can influence insulin receptor phosphorylation, altering its tyrosine kinase activity and therefore inhibiting insulin receptor-initiated signals in hepatocytes [213]. High levels of TNF-α can initiate intracellular signaling which may lead to caspase activation and apoptosis in hepatocytes, casing inflammation and tissue fibrosis [214,215]. Therefore, by reducing TNF-α and inflammation, n-3 PUFA can protect hepatocytes from these damages [214]. The hyperinsulinemia condition that often occurs during IR can affect SREBP-1, a lipogenic transcription factor, which regulates lipid homeostasis by controlling a wide range of enzymes [216], enhancing fatty acid synthesis and accelerating TG accumulation [217].

Zhu et al. [201] conducted a randomized placebo-controlled trial using seal oil as the source of n-3 PUFA in patients with NAFLD associated with hyperlipidemia. Liver steatosis was diagnosed and monitored by US. Oral supplementation of n-3 PUFA (2 g, three times a day) lasted for 24 weeks. No significant changes were observed in body weight and fasting blood glucose. Interestingly, total symptom scores, ALT and TG levels were significantly reduced in the intervention as compared with the placebo group. Moreover, the US revealed that at the end of treatment, 19.70% of the n-3 PUFA supplemented patients showed a normal liver echopattern and that 53.03% of them had an overall fatty liver regression. By contrast, in the placebo group only 7.35% of patients achieved complete regression. Seal oils have a different PUFA composition than fish oils, which have been mainly used in trials in humans. In fishes, EPA and DHA are positioned in sn-2, while in marine mammals, these fatty acids are found mainly at the sn-1 and sn-3 positions of triglycerides. This study suggests that administration of seal oils rich in n-3 PUFA seems to be also efficient in combination with an energy restricted diet in treating patients with NAFLD associated with hyperlipidemia [201].

Sofi et al. [203] conducted a randomized study in subjects with NAFLD (BMI mean of 29.3 kg/m^2^), in which subjects received 6.5 mL/day of olive oil enriched with n-3 PUFA (0.83 g n-3 PUFA, of which 0.47 g was EPA and 0.24 g was DHA), or the same dose of unenriched olive oil for 12 months. No specific diet was recommended, but food habits and estimation of nutrients and calorie intake were recorded; physical activity was also recorded by questionnaires and classified as light or absent, moderate, or intense. After a year of treatment, no significant BMI differences were found, while hepatic and lipid parameters were improved in the subjects receiving the n-3 PUFA-enriched olive oil. Indeed, ALT, AST, and GGT decreased by 40.4%, 35.3% and 27.2% respectively, and serum TG levels reduced by 19.2%. Contrariwise, a predominant increase of HDL-C and adiponectin by 36.1% and 30.2% was observed in the intervention group. Moreover, the echo-Doppler test revealed an increase of 26.7% in DPI in the group consuming the n-3 PUFA-enriched olive oil [203].

The WELCOME study (Wessex Evaluation of fatty Liver and Cardiovascular markers in NAFLD with OMacor thErapy) evaluated whether the supplementation with highly purified n-3 PUFA could exert a more beneficial effect on NAFLD [204]. Overweight/obese patients diagnosed with NAFLD received 4 g/day of placebo (olive oil) or Omacor (1g contains 460 mg of EPA and 380 mg of DHA as ethyl esters) for 15 to 18 months. Liver fat percentage was evaluated by MRI and liver fibrosis by using two histological validated scores, which have different sensitivities in assessing grade of severity in steatosis. Erythrocyte enrichment with EPA+DHA was used to validate the adherence to intervention in the Omacor group and monitor contamination with DHA and EPA in the placebo group. The study revealed that n-3 PUFA supplementation did not improve fibrosis scores, while there was a trend to reduce liver fat in these patients. Regression analysis revealed an independent association between the reduction in the percentage of liver fat and DHA enrichment [204].

Qin et al. [208] carried out a double-blind placebo-controlled trial in patients with NAFLD (BMI 26.0), in which subjects received 4 g/day of placebo (corn oil) or fish oil capsules (with a total daily amount of 728 mg of EPA and 516 mg of DHA) for 3 months. After intervention, the fish oil group exhibited higher circulating levels of EPA and DHA, while a reduction was found in serum levels of glucose, TG, total cholesterol, and apolipoprotein B. Fish oil supplementation also reduced markers of inflammation such as TNF-α, leukotriene B4, and prostaglandin E2, while it increased adiponectin, an anti-inflammatory adipokine. In this study, the authors, did not carry out US or MRI to estimate NAFLD, but several circulating markers of NAFLD were evaluated. After treatment, a significant decrease was induced by fish oil supplementation on ALT, GGT, FGF21, and cytokeratin 18 fragment M30. Increased levels of FGF21 have been found in patients with NAFLD, which is associated with chronic inflammation and considered as a result of a FGF21-resistant state [218].

Recently, Tobin et al. [211] conducted a double-blind randomized placebo-controlled study in subjects with NAFLD to evaluate the efficacy of an n-3 PUFA medical food (omega-3 concentrate MF4637) provided as 3 capsules of 1 g per day (each capsule containing marine-sourced EPA and DHA as ethyl esters, 460 mg and 380 mg, respectively) for 24 weeks. The high concentrate omega-3 intake significantly increased the omega-3 index and absolute values of red blood cells (RBC) EPA and DHA, and decreased the RBC n-6: n-3 PUFA ratio. ALT, AST, and GGT decreased in the placebo group but not in the group with the omega-3 concentrate. Liver fat content evaluated by magnetic resonance imaging-proton density fat fraction (MRI-PDFF) significantly decreased in both groups, but no differences were found between groups.

Li et al. [206] performed a randomized, but not blinded trial in overweight/obese participants diagnosed with NASH. The intervention group received n-3 PUFA treatment (50 mL of PUFA with 1:1 ratio of EPA and DHA in the daily diet), while the control group received saline solution for 6 months. Participants were advised to follow a low-fat and low-carbohydrate diet with moderate physical activity for 30 min for at least 5 times a week. Inflammation and oxidative parameters, C-reactive protein (CRP), MDA, as well as fibrotic parameters, type IV collagen and Procollagen-III-peptide (P-III-P) were significantly higher in patients with NASH. After 6 months of intervention with n-3 PUFA, liver function was generally improved, indicated by a reduction in ALT, AST, TG, total cholesterol, CRP, MDA, and type IV collagen and P-III-P.

On the other hand, Argo et al. [207] carried out a double-blind, randomized, placebo-controlled trial in obese subjects diagnosed with NASH in which the intervention group received 3000 mg/day of n-3 PUFA (each capsule of 1000 mg contained 70% total n-3 PUFA in the form of triglycerides: 35% of EPA, 25% of DHA and 10% of other n-3 PUFA). Patients were advised to follow a hypocaloric diet (500-1000 kcal less than estimated for age- and weight-based basal metabolic rate) with less than 30% of fat content and 150 min of aerobic exercise per week. Serum transaminases, lipid profile, IR, and fasting glucose were determined at the beginning and at the end of the study, liver biopsy and MRI were applied to evaluate liver fat. Although a decrease in n-6/n-3 PUFA ratio in RBC as well as in liver fat reduction were noted in n-3 PUFA-treated patients, no significant changes of NASH were found in any group [207].

A study by Dasarathy et al. [209] was carried out with a double-blind placebo-controlled trial in diabetic patients with NASH diagnosed by liver biopsy. Patients received a placebo (corn oil) or n-3 PUFA (2160 mg of EPA and 1440 mg of DHA daily) for 48 weeks. Outcomes revealed no significant changes in body weight or body composition. Liver steatosis and NAS were improved while lobular inflammation worsened in the placebo group; however, there was no significant change in any of the histological measures in the n-3 PUFA group. These outcomes suggest that n-3 PUFA supplementation did not provide any benefit over the placebo in NASH patients with diabetes. The authors reported that the effects of n-3 PUFA on histology and IR were inferior to the placebo.

Nogueira et al. [210] carried out a double-blind, placebo-controlled clinical trial in patients with NASH. The n-3 PUFA supplemented group received a total amount of 0.945 g of n-3 PUFA (64% ALA, 16% EPA, and 21% DHA) for 6 months. Comparison between the final and basal liver histopathologic scores showed no significant changes between the n-3 PUFA and the placebo groups regarding hepatocellular ballooning, liver steatosis, or fibrosis. Surprisingly, the lobular inflammation was improved in the placebo group in parallel with an increase in plasma EPA and DHA levels, suggesting an off-protocol intake of n-3 PUFAs.

All the previous studies have been carried out with different n-3 PUFA formulations containing both EPA and DHA. Only two studies have analyzed the effects of purified EPA on NASH patients.

Thus, Tanaka et al. [202] carried out a pilot trial to evaluate the efficacy of highly purified EPA (2700 mg/day) on 23 biopsy-proven NASH patients for 12 months. No changes were found in body weight, blood glucose, insulin, or adiponectin. However, circulating levels of ALT were significantly decreased. Post-treatment liver biopsies were obtained from 7 patients, and improved hepatic steatosis and fibrosis, hepatocyte ballooning, and lobular inflammation were found in 6 patients. These findings suggest that highly purified EPA treatment could be efficient for NASH treatment, probably due to its anti-inflammatory and antioxidant properties. To better characterize this finding, Sanyal et al. [205] carried out a multicenter, prospective, double-blind, randomized, placebo-controlled trial with EPA-Ethyl ester (1800 mg/day or 2700 mg/day) for 12 months in 243 patients with diagnosed NASH. The highest dose of EPA reduced the circulating levels of TG, but no significant changes on liver enzymes, IR, adiponectin, keratin 18, CRP, or hyaluronic acid were found after EPA supplementation. Moreover, EPA did not have any relevant effect on histologic features of NASH.

From the eight trials analyzing effects of n-3 PUFA on NAFLD patients, five suggested beneficial effects on some biomarkers of liver steatosis such as a decrease of ALT, AST or GGT, or a reduction of fat liver percentage or biomarkers of metabolic abnormalities associated with NAFLD [111,200,201,203,208]. However, the range of doses used in the studies, the type of formulation of n-3 PUFA (fish oil, seal oil, olive oil enriched with n-3 PUFA), the ratio EPA/DHA, if they are in the form of TG or ethyl esters, etc.), as well as the duration of the trials (3 to 12 months) were widely different among the trials. Therefore, it is difficult to provide a concluding recommendation about the most effective dose and duration of the trial. Moreover, two trials using higher doses of EPA and DHA (> 3 g/day) during a longer period of time (15 to 48 months) did not find any significant changes on serum transaminases or NAFLD activity scores [204,209]. In the same way, a recent trial testing the effects of 3 g of a high concentrate omega-3 medical food (1.38 g EPA and 1.14 DHA) has not found significant differences with the placebo group in NAFLD patients [211]. The inconsistent outcomes of the different trials may be also related with the heterogeneity of participants within the spectrum of NAFLD (lean, overweight, or obese, with or without other metabolic complications such as type 2 diabetes or severe dyslipidemia, as well as the severity of the liver steatosis). Trials also differ in the methodology to evaluate the degree of liver disease in NAFLD and NASH patients.

Six studies have analyzed the potential beneficial effects of n-3 PUFA supplementation on NASH diagnosed patients. Two studies suggested that n-3 PUFA supplementation could be useful for improving NASH [202,206]. However, these studies have some limitations. In one of them, the intervention group received 50 mL of PUFA/day with a ratio 1:1 of EPA:DHA added into the diet, but the composition of the PUFA preparation and the exact amount of EPA and DHA received daily by the patients was not indicated. Moreover, the control group received saline and no other oil with similar calorie supply. Patients of both groups reduced their BMI after the intervention, probably in response to the dietary recommendations and modest physical activity. The decrease in BMI tended to be higher in the PUFA group, and therefore the beneficial effects observed could be secondary to a higher reduction of body weight or fat mass, which was not considered in the interpretation of the data [206]. The other study was a pilot trial without a placebo group, which suggested that the administration of highly purified EPA can improve NASH features [202]. However, a further double-blind placebo-controlled trial providing the same dose of highly purified EPA (2700 mg/day) also for 12 months did not find any significant change in steatosis or fibrosis in NASH patients [205]. Similarly, two double-blind placebo-controlled trials using high doses of n-3 PUFA (≥3000 mg/day) for 12 to 48 months did not observe any significant improvement on the histological features of NASH [207,209]. In the same way, Nogueira et al. [210] described that the supplementation with n-3 PUFAs from a flaxseed/fish oil mixture did not improve liver histology in NASH patients when compared to placebo. Therefore, the effectiveness of n-3 PUFA to attenuate severe NAFLD or NASH markers including liver fibrosis or inflammation is still unclear.

Some studies support differential effects for EPA and DHA [219]. Thus, trials in children and adolescent have suggested that DHA was more effective in reducing liver fat and markers of liver fibrosis [220,221]. Moreover, the study of Scorletti et al. [204] found an independent association between reduced liver fat percentage and erythrocyte DHA enrichment (but not with erythrocyte EPA enrichment). However, there are no trials in adults evaluating the efficacy of highly purified DHA in patients with NAFLD/NASH.

In conclusion, the trials performed to date suggest that n-3 PUFA supplementation maybe effective in the early stages of NAFLD, but not in patients with more severe NAFLD or NASH. Further randomized controlled trials are needed to evaluate the efficacy of DHA on these conditions. Also, it is important to better characterize if the n-3 PUFA supplementation is more effective when combined with a calorie restricted diet or exercise.

## 4. Conclusions and Future Perspectives

Oxidative stress has been related with the development of several age-related conditions including cardiovascular and neurodegenerative diseases, and plays a key role in combination with inflammation and lipotoxicity in the progression of NAFLD to NASH. The outcomes derived from the different trials addressing the potential usefulness of n-3 PUFA supplementation to attenuate oxidative stress are controversial (with beneficial, neutral, or even negative actions depending on the oxidative-stress biomarker measured). Those articles that observed a beneficial effect on oxidative stress suggested that the effect could be related to the immuno-modulatory and anti-inflammatory properties of n-3 PUFA and to their ability to increase antioxidant enzymes, which could contribute to reduce the generation of ROS and other oxidative stress agents. However, the heterogeneity in population participating in the randomized trials, the differences in treatment duration, doses, as well as in methods to assess oxidative stress make difficult to conclude about the effectiveness of n-3 PUFA to reduce oxidative stress during aging.

Concerning NAFLD, the systematic review of randomized controlled trials strongly suggests that n-3 PUFA supplementation may be an effective option to decrease liver fat and circulating enzymes related to liver injury in adult patients with NAFLD. However, the effectiveness of n-3 PUFA to attenuate more severe NAFLD or NASH markers including liver fibrosis is still inconclusive. Therefore, it is difficult to provide a recommendation about the proper doses and formulas (EPA vs DHA or EPA/DHA ratio) to attenuate the progression or to reduce NAFLD/NASH. Large-scale, well-designed randomized controlled trials are needed to better characterize the efficacy of n-3 PUFA for oxidative stress and NAFLD/NASH treatment in adults. Future research should also focus on analyzing the differences in bioavailability and effects of different formulations of n-3 PUFA, including re-esterified TG, ethyl ester, carboxylic acids, and phospholipids.

Another important issue is the evidence about the heterogeneity in the response to n-3 PUFA supplementation within-population. Genetic background may clearly influence this differential responsiveness. For example, PNPLA3 rs738409 underlies the response to a variety of treatments in NAFLD [222]. Consequently, future efforts should be paid to the identification of genetic/epigenetic signatures involved in the response to treatments to warrant a personalized, precise medicine in NAFLD.

Metabolomics and lipidomics studies are also needed to better understand the alterations in the metabolome that occur during aging-related pathologies, including NAFLD, as well as the changes in the metalolomic/lipidomic signatures induced by n-3 PUFA, which could contribute to explain the beneficial actions of these fatty acids through the production of SPMs or other metabolites. In this way a recent study using a proteomic approach in liver biopsias and lipidomic analysis of plasma has found that n-3 PUFA supplementation improve markers of lipogenesis, ER stress and mitochondrial function in patients with NASH [223].

Trials should also consider analyzing gut microbiota changes during the aging process as well as their modulation by dietary factors including n-3 PUFA and to characterize the mechanisms linking the relationship between the modifications of gut microbiota composition and the evolution of oxidative stress and liver pathology.

## Figures and Tables

**Table 1:** NAFLD index and their hallmarks.

NAFLD Index	Predictors	Hallmarks	Interpretation
Fatty liver index (FLI)	Fatty Liver Index (FLI) = e^y^ / (1 + e^y^) × 100. Where y = 0.953 × ln(triglycerides, mg/dL) + 0.139 × BMI, kg/m^2^ + 0.718 × ln (GGT, U/L) + 0.053 × WC, cm − 15.745) [95]	Identified NAFLD and the optimal cut-off point with accuracy.	**FLI < 30**, no FL (with a negative likelihood ratio of up to 0.2); **60 < FLI < 30**, Inconclusive **FLI ≥ 60**, FL present (with a likelihood ratio starting from 4.3)
Lipid accumulation product (LAP)	LAP for men = (WC [cm]–65) × (TG concentration [mmol/L]) LAP for women = (WC [cm] − 58) × (TG concentration [mmol/L]) [96]	Associated with the presence and severity of NAFLD, among young and aged population [97,98]. NOT able to predict liver fat content [99].	The optimal cut-off value for LAP was 31.6 with sensitivity of 88% (95% CI, 77–96%), specificity of 82% (95% CI, 76–87%) for males and with a sensitivity of 66% (95% CI, 52–78%), specificity of 93% (95% CI, 88–96%) for females.
Hepatic steatosis index (HSI)	Hepatic steatosis index (HSI) = 8 × (ALT/AST ratio) + BMI (+2, if female; +2, if diabetes mellitus) [89].	A simple, efficient screening tool for NAFLD, used for selecting individuals for liver ultrasonography [89].	At values of < 30.0 or > 36.0, HSI ruled out NAFLD with a sensitivity of 93.1%, or detected NAFLD with a specificity of 92.4%, respectively [89].
The ZJU (Zhejiang University) index	ZJU index = BMI (Kg/m^2^) + FPG (mmol/L) + TG (mmol/L) + 3 × ALT (IU/L)/AST (IU/L) ratio (+2, if female) [90].	Confirmed to have significance in terms of diagnosing NAFLD [90].	At a value of <32.0, the ZJU index could rule out NAFLD with a sensitivity of 92.2%, and at a value of >38.0, the ZJU index could detect NAFLD with a specificity of 93.4% [90].
NAFLD fibrosis score	NAFLD Score = −1.675 + (0.037 × age [years]) + (0.094 × BMI [kg/m^2^]) + (1.13 × IFG/diabetes [yes = 1, no = 0]) + (0.99 × AST/ALT ratio) − (0.013 × platelet count [×109/L]) − (0.66 × albumin [g/dL]) [92]	Identifies patients without severe fibrosis, comparatively more difficult to estimate [91].	Low cut-off score (−1.455): advanced fibrosis ruled out with high accuracy (negative predictive value of 93% and 88% in the estimation and validation groups, respectively). High cut-off score (0.676), advanced fibrosis diagnosed with high accuracy (positive predictive value of 90% and 82% in the estimation and validation groups, respectively) [92].
BARD score	Based on AST/ALT ratio, presence of diabetes and BMI [100].	Identifies patients without severe fibrosis, but easier to estimate and does not have indeterminate results [91].	BMI ≥28 = 1 point, AAR of ≥0.8 = 2 points, DM = 1 point A score of 2–4 was associated with an OR for advanced fibrosis of 17 (confidence interval 9.2 to 31.9) and a negative predictive value of 96% [100].
FIB-4 index	FIB-4 Score = age ([yr] × AST [U/L])/((PLT [10^9^/L]) × (ALT [U/L])^1/2^) [101].	In patients <35 or >65 years old, the score has been shown to be less reliable [94,101].	At a cut-off of <1.45 in the validation set, the negative predictive value to exclude advanced fibrosis (stage 4–6) was 90% with a sensitivity of 70%. A cut-off of >3.25 had a positive predictive value of 65% and a specificity of 97%. Using these cut-offs, 87% of the 198 patients with FIB-4 values outside 1.45–3.25 would be correctly classified [101].

Abbreviations: AAR, AST/ALT ratio; BMI, body mass index; DM, diabetes mellitus; FPG, fasting plasma glucose; TG, triglycerides; ALT, alanine transaminase; AST, aspartate transaminase; GGT, gamma-glutamyl transpeptidase; OR, odd ratio; WC, waist circumference.

**Table 2:** Effects of n-3 PUFA supplementation in oxidative stress in healthy subjects or in population with cardiometabolic disorders.

Reference	Study Design	Population	Intervention	Outcome Measurements	Comments
Meydani et al., 1991 [170]	Randomized intervention before and after comparison	Young females, aged 51–71; *n* = 14. Old females, aged 51–71. *n* = 9	1680 mg EPA + 720 mg DHA per day for 3 months	Plasma MDA level	↑Plasma MDA level
Harats et al., 1991 [171]	Randomized parallel clinical trial	Study A: Smokers: Control: BMI: 23.5 ± 1.2, age: 42.6, *n* = 5 Fish oil: 23.8 ± 0.8, age: 37.4, *n* = 6 Study B: Smokers Control: BMI: 24.5 ± 1.2, age: 31, *n* = 3 Fish oil: BMI: 25.0 ± 1.3, age: 29.1, *n* = 3 Fish oil + VitE: BMI: 23.5 ± 1.2, age: 35.2, *n* = 4 Study C: Non-smokers, normolipidemic: Control: BMI: 23.7, age: 36.8, *n* = 8 Fish oil: BMI: 24.7 ± 1.3, age: 41, *n* = 6 Fish oil + VitE: BMI: 24.5 ± 1.9, age: 38.8, *n* = 6	Study A: Fish oil: concentrate (MaxEPA), 10 g/d for 4 weeks. Study B and C, Fish oil: (MaxEPA), 10 g/d for 4 weeks Fish oil (10 g/day) + Vit E (400 mg/d) for 4 weeks	Plasma and LDL TBARS level	10 g/d of fish oil consumption ↑plasma LDL TBARS level in smokers and non-smokers Vitamin E counteracted the effect of fish oil more effectively in non-smokers
Nenseter et al., 1992 [176]	Randomized placebo-controlled parallel clinical trial	Normolipidemic subjects Treatment: women and men, BMI not reported, age: 27–63, *n* = 12 Control: women and men, BMI not reported, age: 23–70, *n* = 11	Treatment: 6 g capsules/d of *n*-3 PUFA (highly concentrated ethyl esters). Control: 6 g of corn oil Duration: 4 months	Susceptibility of LDL to Lipid peroxides formation	↔ Lipid peroxides formation
Frankel et al., 1994 [177]	Randomized, double-blind, clinical trial	Hypertriglycemic men and women, age, BMI, smoking status not reported. *n* = 9/group	Control group: fish oil absent from the diet. Supplemented group: 5.1 g of fish oil per day for 6 weeks	LDL oxidative susceptibility	↔ LDL oxidative susceptibility
Brude et al., 1997 [178]	Randomized, double-blind, placebo-controlled parallel clinical trial	Male smokers, hyperlipidemia, aged 40–60, BMI not mentioned. *n*-3 PUFA capsule group (*n* = 11), antioxidant group (*n* = 11), *n*-3 PUFAS + antioxidants group (*n* = 11), control oil group (*n* = 9)	*n*-3 PUFAS group: 5 g DHA and EPA/d Antioxidants capsule, 75 mg Vit E, 150 mg Vit C, 15 mg β-carotene, and 30 mg coenzyme Q10 per day Control group: 8 g of oil with an FA pattern similar to an ordinary Norwegian diet Lasted for 6 weeks	LDL oxidative susceptibility, lipid peroxides	↔ LDL oxidative susceptibility, ↔ lipid peroxides
Mori et al., 1999 [159]	Randomized, controlled parallel study	49 untrained and sedentary NIDDM patients. Age: 30–65 y. BMI < 36 kg/m^2^	Study 1: Group 1: Low-fat diet (30% of daily energy) (*n* = 14) Group 2: Low-fat diet + one daily fish meal (3.6 g n-3 PUFA/day) (*n* = 12) Group 3: Low-fat diet + Moderate exercise (*n* = 11) Group 4: Low-fat diet + Fish meal + moderate exercise (*n* = 12) for 8 weeks.	Urine F2- isoprostanes	Urine F2- isoprostanes
Higdon et al., 2000 [160]	Randomized blinded, crossover study	Post-menopausal women, aged between 50–75, BMI < 30 kg/m^2^, non-smokers, *n* = 15	Fish oil group: 15 g/d (2.0 g EPA/d and 1.4 g DHA/d) Safflower oil group: 15g/d (10.5 g linoleate/d); Sunflower oil: 15g/d (12.3 g oleate/d) in a 3-treatment crossover trial (5 weeks with a 7-wk washout interval)	Plasma F2-isoprostanes, MDA, and TBARS	In fish oil group: ↓plasma F2-isoprostanes ↓MDA ↑TBARS
Wander and Du, 2000 [172]	Randomized crossover study	Post-menopausal women, aged 45–75, BMI < 30 kg/m^2^, smoking status not reported. *n* = 46	Group 1: fish oil (2.5 g EPA and 1.8 g DHA) Group 2: fish oil (2.5 g EPA and 1.8 g DHA) + 100 mg α-tocopheryl acetate Group 3: fish oil (2.5 g EPA and 1.8 g DHA) + 200 mg α-tocopheryl acetate Group 4: fish oil (2.5 g EPA and 1.8 g DHA) + 400 mg α-tocopheryl acetate for 5 weeks (4-period crossover design)	TBARS, protein oxidation	↑TBARS. Protein oxidation not changed
Mori et al., 2000 [162]	Randomized, placebo-controlled parallel study	Overweight, mildly hyperlipidemic men, age: 20–65 y, BMI: 25–30 kg/m^2^	Group 1: 4 g/d of purified EPA (*n* = 19) Group 2: 4 g/d of purified DHA (*n* = 17) Group 3: 4 g/d of olive oil (*n* = 20) for 6 weeks	Urine F2- isoprostanes	↓Urine F2- isoprostanes in the EPA, DHA treatment groups
Wu et al., 2006 [179]	Randomized, single-blind, placebo-controlled parallel clinical trial	Post-menopausal vegetarian women, aged <60. Corn oil: *n* = 13 DHA: *n* = 14	Corn oil group: 6 g corn oil/day DHA-rich algae oil group: 2.14 g of DHA/day for 6 weeks	Plasma α-tocopherol, urine F2-isoprostanes	↔Plasma α-tocopherol, urine F2-isoprostanes
Egert et al., 2007 [173]	Randomized parallel controlled study	Healthy men and women, aged: 25.9 ± 6.82; BMI: 22.2 ± 2.95, non-smokers. *n* = 48	ALA group: Rapeseed oil +1% of energy of ALA (*n* = 15) EPA group: Rapeseed oil + 1% of energy of EPA (*n* = 17) DHA group: Rapeseed oil + 1% of energy of DHA (*n* = 16)	*Ex vivo* LDL oxidative susceptibility	EPA and DHA group: ↑ *ex vivo* LDL oxidative susceptibility
Cazzola et al., 2007 [163]	Randomized parallel placebo-controlled intervention	Healthy young men (age: 14–42 y, BMI: 24.1 ± 0.3). *n* = 93 Healthy old men (age: 53-70 y. BMI: 27.6 ± 0.0). *n* = 62	4 young and 4 older groups: 1: 1.35 g EPA + 0.27 g DHA per day; 2: 2.7 g EPA + 0.54 g DHA per day; 3: 4.05 g EPA + 0.81 g DHA per day; 4: corn oil group Lasted for 12 weeks	Plasma lipid hydroperoxides Lag time of lipoprotein peroxidation	↓Plasma lipid hydroperoxides ↓Lag time of lipoprotein peroxidation and ↓GSH/Gluthatione in olders
Hanwell et al., 2009 [180]	Randomized, double-blind, placebo-controlled crossover clinical trial	Hyper-triglyceridemic, overweight, and obese men; aged > 45, smoking status not reported. *n* = 10 in total	High-fat, high-fructose meal in all groups: Fish oil group: 7 g of fish oil concentrate (2.8 g EPA and 1.4 g DHA) Isoflavone group: 336 mg NovaSoy (150 mg glycoside isoflavones). Fish oil + isoflavone: 7 g fish oil + 336 mg NovaSoy Placebo group: 7 g corn oil Consumed 4 days separated by 1week wash out.	Lipid peroxides, oxidized LDL, total antioxidant status	↔ Lipid peroxides, ↔ oxidized LDL, ↔ total antioxidant status
Bloomer et al., 2009 [174]	Randomized, double-blind crossover study	Subjects are exercise trained man, non-smokers, no history of cardiometabolic diseases. Age: 25.5 ± 4.8 y. BMI: 24.1 ± 1.6 *n* = 14	Intervention group: 2.224 g EPA and 2.208 g DHA per day Control group: same quantity of soybean oil Duration: 6 weeks (with 8-week washout) Supplementation were prior to performing a 60 min treadmill climb using a weighted pack	Blood was collected pre and post exercise and analyzed for a variety of oxidative stress (Protein carbonyls, IgG-autoantibodies, low-density lipoprotein, Malondialdehyde, Hydrogen peroxide and xanthine oxidase activity, Nitric oxide, Whole blood lactate and inflammatory biomarkers	Resting levels: ↓ CRP, ↓TNF- α, ↔ MDA, ↔ Nitric oxide. Exercise: ↑ oxidative biomarkers (mild)
Mas et al., 2010 [164]	Randomized, Placebo-controlled intervention	Study A: placebo-controlled intervention (BMI: 25–30), dyslipidemic men, age: 20–54 y, *n* = 17–20 per group. Study B: hypertensive type 2 diabetic and post-menopausal women, age: 40–75 y, *n* = 16–18	In both studies, n-3 PUFA group: 4 g/day of EPA or DHA Control group: Olive oil placebo lasted for 6 weeks	Plasma F2-isoprostanes	↓ Plasma F2-isoprostanes with *n*-3 PUFAS supplementation
Petersson et al., 2010 [181]	Randomized parallel study	Participants with metabolic syndrome, age: 35–70 y, BMI: 20–40 kg/m^2^, smokers or non-smokers. Saturated high-fat diet: *n* = 100 Monosaturated high-fat diet: *n* = 111 Low-fat diets with n-3 PUFA: *n* = 100 Low-fat diets with sunflower oil: *n* = 106	Saturated high-fat diet (38% E fat): (HSFA: 16% SFA, 12% MUFA and 6% PUFA), Monosaturated high-fat diet (38% E fat): (HMUFA: 8% SFA, 20% MUFA and 6% PUFA) Low-fat (28% E) high-complex carbohydrate diets (LFHCC: 8% SFA, 11% MUFA and 6% PUFA) with 1.24 g/d n-3 PUFA Low-fat (28% E)-high-complex carbohydrate diets (LFHCC: 8% SFA, 11% MUFA and 6% PUFA) with 1g/d high-oleic acid sunflower oil For 12 weeks	Urinary levels of 8-iso-PGF2α and 15-keto-dihydro-PGF2α Serum CRP	↔ 8-iso-PGF2α ↔ 15-keto-dihydro-PGF2α ↔ Serum CRP
Ulven et al., 2011 [182]	Randomized parallel study	Participants with normal or slightly elevated total blood cholesterol and/or triglyceride levels, age: 30–50 y, BMI > 30 kg/m^2^	Krill oil group: 3 g/day (EPA + DHA= 543 mg/day) in 6 capsules (*n* = 36) Fish oil group: 1.8 g/day (EPA + DHA= 864 mg/day) in 3 capsules (*n* = 40) Control group: no supplementation (*n* = 37) Duration: 7 weeks	Urine F2-isoprostanes, plasma α-tocopherol	↔ Urine F2-isoprostanes, ↔ plasma α-tocopherol
Egert et al., 2012 [184]	Randomized single-blind parallel	Men and premenopausal women; Age: 19–43 y; BMI < 28 kg/m^2^, non-smokers	Margarines fortified with 10% weight of EPA, DHA, or ALA EPA group: 2.2 g/day (*n* = 25). DHA group: 2.3 g/day (*n* = 25) ALA group: 4.4 g/day (*n* = 24) For 6 weeks	Antioxidant capacity, plasma MDA, RBC-MDA, linoleic acid hydroperoxides (LA-OOH) in RBC	↔ Antioxidant capacity ↑Plasma MDA in EPA and DHA groups. ↔ RBC-MDA ↓ RBC-LA-OOH
Kirkhus et al., 2012 [185]	Open, randomized parallel study	159 healthy men and women. Age: 18–70 y, BMI < 30 kg/m^2^, moderate smokers	1g/day of EPA + DHA as: - fish pâté (34 g). *n* = 44 - n-3 PUFA-enriched fruit juice (500 mL). *n* = 38 - 3 capsuled of fish oil. *n* = 40 - Control: non-supplemented. *n* = 37 Duration: 7weeks	Urine F2-isoprostanes and plasma α-tocopherol	↓Plasma α-tocopherol in fish pâté group when calculated in relation to the level of serum TG ↔ F2-isoprostanes
Ottestad et al., 2012 [183]	Randomized, double-blind, placebo-controlled parallel study	54 Healthy men and women, age: 18–50 y, BMI < 30 kg/m^2^, non-smokers	Group 1: 8 g/d of fish oil (EPA/DHA) *n* = 17 Group 2: 8 g/d of oxidized fish oil (EPA/DHA) *n* = 18 Group 3: 8 g/d of high-oleic sunflower oil *n* = 19 For 7 weeks	Urine F2-isoprostanes and plasma oxidation products from n-3 PUFA and n-6 PUFA oxidation 4-HHE and 4-HNE; plasma α-tocopherol, enzymatic activity of GR, GPx, and CAT	↔ Urine F2-isoprostanes and ↔ plasma oxidation products from n-3 PUFA and n-6 PUFA oxidation ↔ 4-HHE and ↔ 4-HNE; ↔ plasma α-tocopherol, ↔ enzymatic activity of GR, GPx, and CAT
Schimidt et al., 2012 [165]	Randomized, controlled, parallel intervention	10 normo and 10 dyslipidemic men; Age: 29–51, BMI: 35 kg/m^2^. *n* = 20	6 Fish oil capsules, providing 1.14 g DHA and 1.56 g EPA per day, for 12 weeks	GST, GR, and antioxidative enzymes SOD3, CAT, and HMOX2 expression in whole blood cells, GPx, MMPs, cyrochrome P450 (CYP) enzymes expression in whole blood	↑GST, ↑GR and antioxidative enzymes ↑SOD3, ↑CAT and HMOX2 expression, ↓GPx, ↓MMPs, ↓cytochrome P450 (CYP) enzymes expression
Kiecolt-Glaser et al., 2013 [166]	Randomized, double-blind, controlled parallel trial	Healthy sedentary overweight middle-aged and older adults Age: 48–85 y, BMI: 22.5–40 kg/m^2^. Non-smokers	Group 1: 2.5 g/day n-3 PUFA (*n* = 35), Group 2: l.25 g/day n-3 PUFA (*n* = 40) Group 3: placebo capsules that mirrored the proportions of fatty acids in the typical American diet (*n* = 31) Duration: 4 months	Plasma F2-isoprostanes	↓Plasma F2-isoprostanes with n-3 PUFAS supplementation
Haijianfar et al., 2013 [167]	Randomized double-blind placebo-controlled clinical trial	Type 2 diabetic women. Age: 45–65 y BMI: 27.7 ± 3.4 (n-3 PUFA group). BMI: 28 ± 3.8. (Control group)	n-3 PUFA group: 2000 mg/d in 2 capsules: each contained 1,000 mg n-3PUFA (65% EPA, 360 mg and 35% DHA, 240 mg) (*n* = 37) Control group: 2 placebo capsules, each contains 1 g of cornstarch (*n* = 34) Duration: 8 weeks	Serum antioxidant capacity	↑Antioxidant capacity in the n-3 PUFA supplemented group
Véricel et al. 2015 [168]	Randomized, double-blind, placebo-controlled, two-period crossover trial	Post-menopausal women with type 2 diabetes, age: 59.8 ± 4.7 y, BMI: 34.1 ± 5 kg/m^2^. *n* = 11	Intervention: 400 mg/day of DHA (in 2 capsules/d) Control: 2 placebo (same amount of sunflower oil) Duration: 2 weeks	Plasma and platelet vitamin E, alpha- and gamma-tocopherol concentrations, plasma MDA, 8-iso-PGF2α	↑ Platelet alpha-tocopherol, gamma-tocopherol tend to increase. ↓MDA, ↓8-iso-PGF2α. n-3 PUFAS supplementation ↓ oxidative stress associated with diabetes
Alves Luzia et al. (2015) [175]	Randomized, double-blind, placebo-controlled trial	Women (40 to 70 years) with low habitual fatty fish and seafood intake, who met at least two of the following criteria: total cholesterol > 200 mg/dL, LDL-C > 140 mg/dL, HDL-C < 50 mg/dL, and triglycerides >150 mg/dL	The fish oil group: daily consumption of 1 g n-3 PUFA (540 mg EPA + 360 mg DHA) and 1 capsule of placebo (*n* = 22) Fish+VitE group: 1 g n-3 PUFA, 400 mg vitamin E/ alpha-tocopherol (*n* = 19). Placebo group: 2 capsules/d mineral oil (*n* = 18) Duration: 3 months	Biomarkers of oxidative stress at baseline, 45 and 90 days	↑ TBARS in the group supplemented with fish oil alone, but not in the fish oil + vitamin E group
Berge et al., (2015) [169]	Randomized, clinical interventional pilot study	Healthy female and male, mean age: 23 ± 4 y. BMI: 20.9 kg/m^2^, n=17	17 subjects received dietary supplementation with krill oil (832.5 mg EPA and DHA per day) for 28 days	Plasma total antioxidant capacity (AOC)	↑AOC after krill oil intake. AOC positively correlated with plasma EPA concentration and RBC EPA concentration
Fayh et al., 2018 [161]	Randomized, double-blind, placebo-controlled trial	Male and female with T2DM, Age: 50–57 y. Mean BMI: 28.2 kg/m^2^ in n-3 PUFAS group and 28.8 kg/m^2^ in control group. *n* = 15/group	Control group: 3 capsules/day that contains 500 mg gelatin Intervention group: 3 capsules/d (each capsule contains 180 mg EPA, 120 mg DHA, 2 mg Vit E) For 8 weeks At the beginning and at the end of protocol, an acute exercise was performed (treadmill)	TBARS; Plasma F2-isoprostanes, TRAP, SOD activity, hs-CRP	n-3 PUFA supplementation: ↓ TG, ↓TRAP levels after exercise, without a significant effect on inflammatory and oxidative-stress markers

Abbreviations: NAFLD, non-alcoholic fatty liver disease; AST, aspartate transaminase; ALT, alanine transaminase; GGT, gamma-glutamyl transpeptidase; ↑, increased; ↓, decreased; ↔, not changed; TG, triglycerides; US, ultrasonography; DPI, doppler perfusion index; TNF-alpha, tumor necrosis factor-alpha; HOMA-IR, homeostasis model assessment-estimated IR; HDL, high density lipoprotein; FBG, fasting blood glucose; NS, not significant; CRP, C-reactive protein; MDA, malondialdehyde; EPA-E, ethyl-eicosapentanoic acid; MRI, magnetic resonance imaging; NAS, NASH activity score; ApoB, apolipoprotein B, FGF21, fibroblast growth factor 21; PGE2, prostaglandin E2; Hb1C, Hemoglobin A1c.

**Table 3:** Effects of n-3 PUFA supplementation in NAFLD (non-alcoholic fatty liver disease) and NASH (non-alcoholic steatohepatitis) adults.

Reference	Study Design	Population	Intervention	Outcome Measurements	Results	Comments
Capanni et al., 2006. [200]	Open-label trial	Patients with NAFLD proven by US; Age range: 31–77 y. Mean BMI: 28.5 kg/m². *n* = 56	Oral intake of n-3 PUFA (EPA and DHA in a 0.9/1.5 ratio), 1 g capsule a day for 12 months. Intervention group (*n* = 42) *vs* control group (*n* = 14)	Hematochemical tests; Liver fat changes detected by US and liver eco-texture measured by Duplex Doppler US and DPI follow up	↓ AST, ALT, GGT; ↓ fasting TG and glucose ↓arachidonate ↓n-6/n-3 ratio Significant beneficial effects on liver US pattern and ↑ DPI	Long-term n-3 PUFAS supplementation ameliorates hepatic steatosis in NAFLD patients
Spadaro et al., 2008. [111]	Randomized open-label trial	Patients with NAFLD proven by US; Mean age: 51 y Mean BMI: 30.5 kg/m²	AHA diet + 2 g/d n-3 PUFA (group DP, *n* = 20) AHA diet (Group D, *n* = 20) for 6 months	Changes on liver fat via US; ALT, AST, GGT, lipid profile, TNF-α serum levels, fasting glucose, and IR by HOMA-IR	Group DP: ↓ ALT, GGT ↓ TG, TNF-α ↓ HOMA-IR ↑ HDL cholesterol Complete steatosis regression in 33.4 % of patients and an overall reduction of 50%.	n-3 PUFA have a major improvement on fatty liver in patients with NAFLD
Zhu et al., 2008. [201]	Randomized controlled trial	Patients with US proven NAFLD associated with hyperlipidemia; Age: 18–65 y	Oral supplementation of n-3 PUFA for 24 weeks. AHA-based diet with a caloric restriction of 25-30 kcal/kg per day Group A (*n* = 66): 2 g n-3 PUFA from seal oils, 3 times/day. Group B (*n* = 68) 2 g placebo, three times/day	Primary endpoints: fatty liver assessed by symptom scores, ALT and serum lipid levels at 8, 12, 16, and 24 weeks. Secondary endpoints: liver fat changes by US at weeks 12 and 24	After 24 wk of treatment: ↔ body weight, ↔ FBG; ↓ Total symptom scores, ↓ALT and TG Complete fatty liver regression was observed in 19.7% of the patients, and an overall reduction was found in 53.0% (35/66) of the patients in group A	n-3 PUFA from seal oils is safe and efficacious for patients with NAFLD associated with hyperlipidemia and can improve their total symptom scores, ALT, serum lipid levels, and normalization of ultrasonographic evidence
Tanaka et al., 2008. [202]	Pilot Trial	23 biopsy-proven NASH patients	Highly purified EPA (2700 mg/d) was administered for 12 months	Biochemical parameters of glucose and lipid metabolism, inflammatory and iron metabolism oxidative-stress markers Ultrasonography Histologic evaluation of liver biopsies	↓ ALT, AST ↓Total cholesterol ↓ sTNFR1,2 ↓Ferritin ↓ Thioredoxin ↓ hepatic steatosis and fibrosis, hepatocyte ballooning, and lobular inflammation	EPA treatment seems to be safe and efficacious for patients with NASH
Sofi et al., 2010. [203]	Randomized	Patients with NAFLD proven by US. Age: 30–70 y, mean BMI: 29.3 kg/m²	Food consumption enriched with n-3 PUFA (0.47 g EPA + 0.24 g DHA) for 12 months. Group 1: (*n* = 6) 6.5ml/d enriched with olive oil + recommended diet Group 2: (*n* = 5) control (recommended diet + not enriched olive oil)	Liver eco-texture measured by Duplex Doppler US and DPI. Liver enzymes, TG and adiponectin levels	↓ ALT, AST, and GGT ↓TG level ↑HDL cholesterol ↑adiponectin ↑ DPI level	Persistent consumption of food enriched with n-3 PUFA has favorable effects in patients with NAFLD
Scorletti et al., 2014. [204]	WELCOME study: double-blind, randomized, placebo-controlled trial	Patients with histological confirmation of NAFLD. Mean age: 50 years old. Mean BMI: 32.5 kg/m²	Intervention group (*n* = 51): oral supplementation of purified long-chain n-3 PUFA ethyl esters (1 g contains 460 mg of EPA and 380 mg of DHA), 4 g/day. Placebo group (*n* = 52): 4 g/day of olive oil. 15 to 18 months of treatment	Liver fat percentage assessed by MRS and biomarker scores for liver fibrosis, erythrocyte enrichment quantification with DHA+EPA via gas chromatography	Trend to improve liver fat% with DHA+EPA No improvement in liver fibrosis scores	Association between erythrocyte DHA enrichment with DHA+EPA treatment and a decrease of liver fat percentage
Sanyal et al., 2014. [205]	Double-blind, randomized, placebo-controlled trial	Patients with NASH, NAFLD activity scores ≥ 4, with minimum scores of 1 for steatosis and inflammation, along with either ballooning or at least stage 1a fibrosis. *n* = 243	Subjects were randomly assigned to groups given placebo (*n* = 75), low- dosage EPA-E (1800 mg/d; n = 82), or high-dosage EPA-E (2700 mg/d; *n* = 86) for 12 months	The primary end point: NAFLD activity score ≤3, without worsening of fibrosis; or a decrease in NAFLD activity score by ≥2 with contribution from >1 parameter, without worsening of fibrosis. Liver enzymes, IR, adiponectin, keratin 18, hs-CRP, or hyaluronic acid were measured as well	No effects of EPA-E on steatosis, inflammation, ballooning, or fibrosis scores. No effects on levels of liver enzymes, IR, adiponectin, keratin 18, hs-CRP, or hyaluronic acid. High-dosage EPA-E: ↓ levels of TG	In a phase 2 trial, EPA-E had no significant effect on the histologic features of NASH. EPA-E reduced subjects’ levels of triglyceride compared with placebo, without any increase in serious adverse events
Li et al., 2015. [206]	Randomized placebo-controlled trial	Patients diagnosed with NASH Mean age: 51 years old. Mean BMI: 27.9 kg/m²	Intervention group (*n* = 39): 50 mL of PUFA with 1:1 Ratio of EPA and DHA added into daily diet placebo: saline (*n* = 39). Duration of treatment: 6 months	Liver enzymes, lipid profile, markers of inflammation and oxidation, and histological changes by biopsy	Liver function was significantly improved: ↓ ALT / AST ↓ TG ↓Total Cholesterol ↓ CRP (inflammation) ↓ MDA (oxidation) ↓ fibrotic parameters	6 months of n-3 PUFA therapy is beneficial for improving NASH
Argo et al., 2015. [207]	Double-blind, randomized, placebo-controlled trial	Patients 34 subjects with biopsy-proven NASH; Mean age: 47 y Mean BMI: 32.5 kg/m^2^	Oral supplementation of n-3 PUFA 3000 mg/d (each 1000 mg capsule contains 35% EPA, 25% DHA and 10% other n-3 PUFA), vs placebo (soybean oil). *n* = 17 per group 1 year of treatment	Liver biopsy, Abdominal MRI for quantitative assessment of hepatic fat, AST, ALT, total cholesterol, LDL and HDL cholesterol, and TGs. FFAs, insulin, and glucose levels	No differences for the primary end point of NASH activity score (NAS) reduction. In n-3 PUFA-treated subjects: ↓in liver fat content by MRI (among subjects with increased or stable weight)	Treatment did not exert beneficial effects towards hepatic histological improvement in NASH patients
Qin et al., 2015. [208]	A double-blind randomized Placebo-controlled clinical trial	Patients with NAFLD associated with hyperlipidemia, Mean age: 44.3 ± 10.9 and 46.0 ± 10.6 y for placebo or treated group Mean BMI: 26.0 ± 2.8 and 26.4±3.9 kg/m², respectively. *n* = 70	Randomly assigned to consume fish oil (*n* = 36, 4 g/d) or corn oil capsules (*n* = 34, 4 g/d) for 3 months	Blood levels of lipids, glucose and insulin, liver enzymes, and cytokines at baseline and the end of the study were measured	Fish oil group: ↓ total cholesterol, ↓ TG, ↓apolipoprotein B ↓ glucose, ↓ ALT ↓ GGT ↑ Adiponectin ↓ TNF-α ↓ LTB4, ↓ FGF21, ↓ CK-18/M30 ↓PGE2	These findings suggest that fish oil can benefit metabolic abnormalities associated with NAFLD
Dasarathy et al., 2015. [209]	Double-blind, randomized, placebo-controlled trial	Patients with NAFLD and NASH diagnosed by liver biopsy, Mean age: 50 y. Mean BMI: 35 kg/m². *n* = 37	n-3 PUFA group: oral supplementation of 2160 mg of EPA and 1440 mg of DHA. (*n* = 19) and Placebo group (*n* = 18) using corn oil supplementation Duration: 48 months	Primary endpoints: assess the improvement of 2 points in the NAFLD activity score by liver biopsy. Secondary endpoints: changes in liver enzymes, IR, fasting glucose, and HbA1C	No differences between groups in BMI, serum transaminases, diabetes control, histological evaluation of NAFLD activity score and individual components	N-3 PUFA supplementation showed no beneficial effects in NASH patients with diabetes
Nogueira et al., 2016 [210]	Double-blind, randomized, placebo-controlled trial	Men and women with a proven histological diagnosis of NASH. Mean age: 53.9 ± 1.8 and 52.5 ± 7.2 y for placebo group and n-3 PUFA group. Mean BMI: 30.3 ± 4.4 and 31.1 ± 4.6, respectively. *n* = 50	n-3 PUFA group: 3 capsules (0.945 g in total per day, 64% ALA, 16% EPA, and 21% DHA). (*n* = 27) Placebo group: 3 capsules of mineral oil (n = 23). Duration: 6 months	Primary endpoints: Plasma fatty acids (ALA, EPA, DHA and AA), NAS. Secondary endpoints: serum TG, AST, ALT, GGT, fasting lipid profile, fasting glucose, anthropometric parameters, or plasma levels of IL-6 at baseline and at endpoint,	n-3 PUFA group: ↑plasma ALA and EPA. NAS correlated with↑plasma ALA. ↓TG Control group: ↑plasma DHA and EPA, NAS correlated with↑plasma DHA and EPA	No significant changes were observed on liver histology in the n-3 PUFA or placebo group
Tobin et al., 2018 [211]	Double-blind, randomized, placebo-controlled trial	Patients with previously diagnosed NAFLD (hepatic steatosis stage). Mean age; 55.1 ± 10.9 and 55.3 ± 13.3 y for placebo and n-3 PUFA MF4637 group Mean BMI; 32.4 ± 5.0 and 32.1±4.8. respectively. *n* = 176	n-3 PUFA group: oral supplementation of 3g capsule (1380 g of EPA and 1140 g of DHA) (*n* = 87) Placebo group: oral supplementation of 3 g olive oil capsule (*n* = 89) Duration = 24 weeks	n-3 PUFA index, n-6 PUFA: n-3 PUFA ratio, quantitative measurements of RBC EPA and DHA at the baseline and the endpoint, liver fat content measured by MRI	n-3 PUFA group: ↑n-3 PUFA index and ↑absolute values of RBC EPA and DHA, ↓RBC n-6: n-3 ratio ↓liver fat content in both groups	No significant differences in fat liver were found between n-3 PUFA and placebo group

**Abbreviations:** NAFLD, non-alcoholic fatty liver disease; AST, aspartate transaminase; ALT, alanine transaminase; GGT, gamma-glutamyl transpeptidase; ↑, increased; ↓, decreased; ↔, not changed; TG, triglycerides; Chol: cholesterol; US, ultrasonography; DPI, doppler perfusion index; TNF-α, tumor necrosis factor-alpha; HOMA-IR, homeostasis model assessment-estimated IR; HDL, high density lipoprotein; FBG, fasting blood glucose; NS, not significant; CRP, C-reactive protein; MDA, malondialdehyde; EPA-E, ethyl-eicosapentanoic acid; MRI, magnetic resonance imaging; NAS, NASH activity score; ApoB, apolipoprotein B, FGF21, fibroblast growth factor 21; PGE2, prostaglandin E2; HbA1C, Hemoglobin A1c; RBC, red blood cells; MRI, magnetic resonance imaging.
